# G1405 Ribosomal Methyltransferase-Driven Antibacterial Resistance Affects the 4,5-Disubstituted-2-deoxystreptamine Class of Aminoglycoside Antibiotics

**DOI:** 10.1021/jacsau.5c01358

**Published:** 2026-02-02

**Authors:** Sven N. Hobbie, Andrea Vasella, Erik C. Böttger, David Crich

**Affiliations:** a Institute of Medical Microbiology, Universität Zürich, Gloriastrasse 30, Zürich CH-8006, Switzerland; b Organic Chemistry Laboratory, ETH Zürich, Vladimir-Prelog-Weg 1-5/10, Zürich 8093, Switzerland; c Innovations in Drug Discovery Program, Department of Pharmaceutical and Biomedical Sciences, 1355 University of Georgia, 250 West Green Street, Athens, Georgia 30602, United States; d Department of Chemistry, 1355 University of Georgia, 302 East Campus Road, Athens, Georgia 30602, United States; e Complex Carbohydrate Research Center, 1355 University of Georgia, 315 Riverbend Road, Athens, Georgia 30602, United States

**Keywords:** aminoglycoside antibiotics, antibacterial resistance, ribosomal methyltransferases, multidrug resistance, apramycin

## Abstract

The 4,5-disubstituted-2-deoxystreptamine (DOS) aminoglycosides (AGAs) and the 4-monosubstituted DOS AGA apramycin have long been known not to be affected by N7 methylation of the 16S rRNA base G1405, a critical mechanism of aminoglycoside resistance caused by ribosomal methyltransferases (RMTases). This puts the 4,5-AGAs and apramycin in a class apart from the 4,6-AGAs, whose action is blocked by RMTase-mediated G1405 N7 methylation and has rendered them attractive candidates for modification in drug-discovery campaigns. Contrary to this common perception, we reveal that multiple modifications of the 4,5-AGAs result in compounds whose minimum inhibitory concentrations are affected by G1405 N7 ribosomal methyltransferases. We argue that the combination of destabilization of the drug-ribosome complex caused by drug modification and G1405 N7 methylation, each of which alone may be insufficient to negatively impact activity, can result in reduced antibacterial activity. In contrast, AGA modifications that enhance affinity for the drug binding pocket will afford compounds that are not susceptible to G1405 RMTase activity, as is found for propylamycin and the apralogs. Future antibiotic discovery campaigns based on 4,5-AGAs and apramycin should take these findings into account.

## Graphical abstract

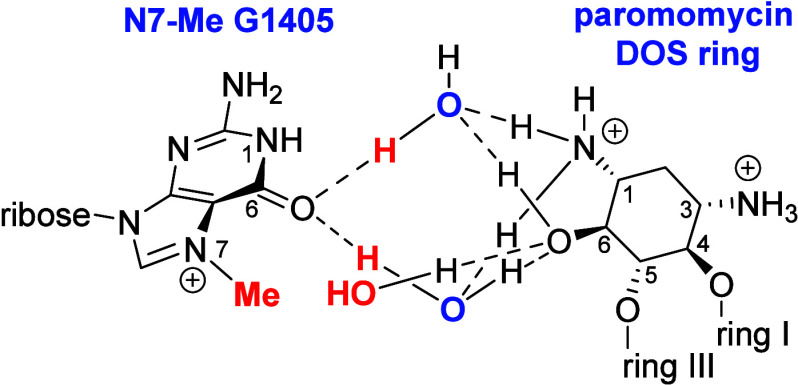

## Introduction

The slow but inescapable spread of multidrug-resistant (MDR) infectious diseases with the potential for outbreaks of pandemic proportions drives the continual need for the development of new anti-infective agents and for new and improved generations of existing drug classes.^1–6^ In our laboratories, we have focused on the development of next-generation aminoglycoside antibiotics (AGAs) driven by (i) the potent broad-spectrum activity of earlier generations, (ii) their lack of allergic reactions, (iii) the extensive existing knowledge of their modes of action, toxicity, and chemical reactivity, and (iv) their widespread availability.^7–21^ In our AGA program, we have focused primarily on the dual goals of overcoming resistance due to the presence of the aminoglycoside-modifying enzymes (AMEs) and of minimizing drug-induced hearing loss, or ototoxicity, which affects a significant proportion of the patient population following treatment with the current generations of AGAs.^7–21^ Here, we turn our attention to the ribosomal methyltransferases (RMTases), a group of *S*-adenosyl-l-methionine-dependent methyltransferases, some of which confer resistance to ribosome-targeting antibiotics through a steric block to drug binding wrought by methylation of nucleotide bases in the drug binding site.^2,7,8,22–25^ For the DOS class of AGAs, whose action derives from binding to the decoding A site on the 30S ribosomal subunit, RMTases from AGA-producing actinomycetes that methylate N1 of A1408 or N7 of G1405 have long been known^26^ but were first detected outside of AGA-producing actinobacteria in 2003.^27,28^ RMTases that act on G1405 N7 block the activity of all 4,6-disubstituted DOS AGAs (4,6-AGAs), including the most recently approved plazomicin,^29–31^ owing to the loss of the hydrogen bond from the 4,6-AGA ring III to G1405 N7 and a steric clash with the methyl group. It has long been reported, however, that G1405 N7 RMTases do not cause resistance to the 4,5-disubstituted DOS AGAs (4,5-AGAs) or to the monosubstituted DOS AGA apramycin because of the absence of a direct hydrogen bond to G1405 N7 from such AGAs.^26,27,32^ The acquisition of A1408 N1 RMTases, on the other hand, results in resistance to all DOS-type AGAs with the exception of some gentamicin isomers,^26,27,32,33^ because both the 4,5- and 4,6-AGAs and apramycin all engage in a doubly hydrogen-bonded ″pseudobase pair” with A1408.

X-ray crystal structures of A1408 N1 and G1405 N7 RMTases in complex with the 30S ribosomal subunit have been solved,^34^ and in combination with a high throughput screening approach have led to the discovery of a first-in-class small molecule A1408 N1 RMTase inhibitor.^35^ Fortunately, although spreading, A1408 N1 RMTases are still rare. The G1405 N7 RMTases, however, are much more widespread with significant clinical prevalence across the most relevant Gram-negative pathogens including *Acinetobacter baumannii* , *Pseudomonas aeruginosa* , *Klebsiella pneumoniae* , *Escherichia coli* , and *Enterobacter* spp.^22,23,25^ When found on the same genetic element as other antibacterial resistance determinants such as the New Delhi metallo-β-lactamase,^31^ RMTases contribute significantly to multidrug resistance to critical standard-of-care therapeutics.^22,23,25^

Work in our and other laboratories on the development of next-generation AGAs for the treatment of MDR Gram-negative infections has focused on the 4,5-AGAs and the apramycin series in order to avoid inactivation by G1405 RMTase activity.^36–50^ As a part of this effort, we observed that the minimum inhibitory concentrations (MICs) of some 4,5-AGA modifications are unexpectedly affected by RMTase-mediated G1405 N7 methylation and present here our analysis of this observation.

## Results

We screened a series of 12 neomycin, 12 paromomycin, 2 ribostamycin, and 8 apramycin derivatives along with the parent compounds and gentamicin and kanamycin A from the 4,6-AGA series as comparators against *E. coli* engineered strains carrying either ArmA or RmtB RMTases. These strains have been used previously as a source of methylated ribosomes for cell-free translation assays^51^ and in the phenotypic profiling of synthetic analogs.^37,40,52^ As differences in promoter strength and plasmid copy number may lead to different *armA* expression levels in the two sets of *E. coli* strains employed, the expression of *armA* in the recombinant strains was checked by MIC testing against a series of unmodified aminoglycosides (Table 1). We then calculated relative susceptibility ratios by dividing the MIC of compounds against the RMTase strain by the MIC against the isogenic wild-type (WT) reference strain (Table 1). In addition to conferring high-level resistance to 4,6-AGAs, identical susceptibility ratios for both MM294 ArmA and DH5α ArmA strains were obtained for 4,5-AGAs, indicating functional equivalence of the recombinant strains used and that the isogenic background of the strains has little or no influence on the data. Susceptibility ratios between 0.5 and 2 were generally considered within technical variance and not necessarily of biological relevance. As expected, the antibacterial activity of the 4,6-AGAs **1** and **2** was greatly suppressed by the presence of either ArmA or RmtB, while, consistent with previous reports,^26,27,32^ the parent 4,5-AGAs showed little or no susceptibility to these resistance determinants (Table 1).

**Table 1:**
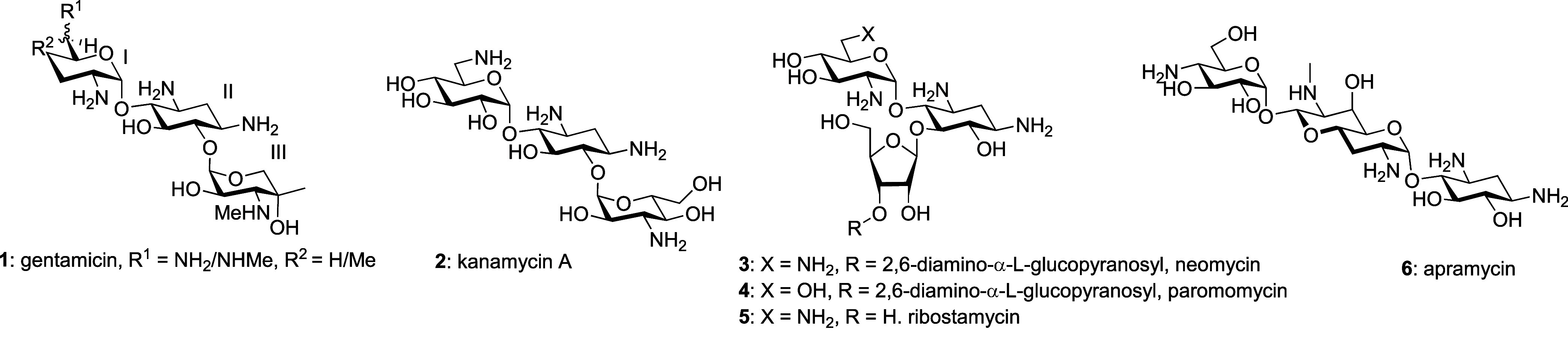
Influence of ArmA and RmtB on the Minimum Inhibitory Concentrations (μg/mL) of Natural 4,6- and 4,5-Aminoglycosides and Apramycin^a^

		*E. coli* MM294			*E. coli* DH5α			susceptibility ratios			
AGA	class	WT^b^	ArmA		WT^b^	ArmA	RmtB		MM294	DH5α	
gentamicin **1**	4,6-	≤0.5	>128		0.5	>64	>64		>256	>128	
kanamycin A **2**	4,6-	0.5–1	>128		0.5	>256	>256		>128	>512	
neomycin **3**	4,5-	0.5	0.5		1	1	1		1	1	
paromomycin **4**	4,5-	2	4		1	1	2		1–2	1–2	
ribostamycin **5**	4,5-	nd	nd		1–2	2–4	2–4			2	
apramycin **6**	4-	nd	nd		2	1	1			0.5	

a nd = not determined.

b WT refers to strains MM294 and DH5α, respectively, containing the plasmid vector without the RMTase insert.

We next investigated a series of neomycin derivatives carrying substituents at either the 2′-, 4′-, or 6′-positions in ring I and at the 5′′-position in ring III that had been synthesized^41–43^ to block the activity of various AMEs. Modification of the neomycin 2′-amino group by either *N*-methylation, conversion to a hydroxy group, or removal has little or no effect on activity against the wild type and a small but consistent effect on the activity of the ArmA strain (Table 2, entries 1–3). Ethylation of the 4′-hydroxy group causes no loss of activity against the wild type but a 4-fold drop in activity in the presence of ArmA (Table 2, entry 4). Introduction of a 2-hydroxyethyl moiety onto the 6′-amino group does not cause any loss of activity against the wild type but causes a 4-fold lower activity in the presence of ArmA, whereas conversion of the 6′-amino group into an acetamide causes a 4-fold loss of activity against the wild type and an even more significant loss of activity in the presence of ArmA resulting in a susceptibility ratio of 8 (Table 2, entries 5 and 6). Combining both the 4′-*O*-ethylation and 6′-*N*-hydroxyethylation into a single compound **13** results in a 4-fold loss of activity against the wild type but a 32-fold loss of activity in the presence of ArmA (Table 2, entry 7). Replacement of the 5′′-hydroxy group of neomycin by an amino or amido group, or indeed deletion of the 5′′-hydroxy group or even the entire hydroxymethyl side chain from the 4′′-position did not occasion any loss of activity against the wild type, but resulted in a 2- to 8-fold loss of activity, depending on the modification, against the ArmA and/or RmtB containing strains, overall (Table 2, entries 8–12).

**Table 2:**
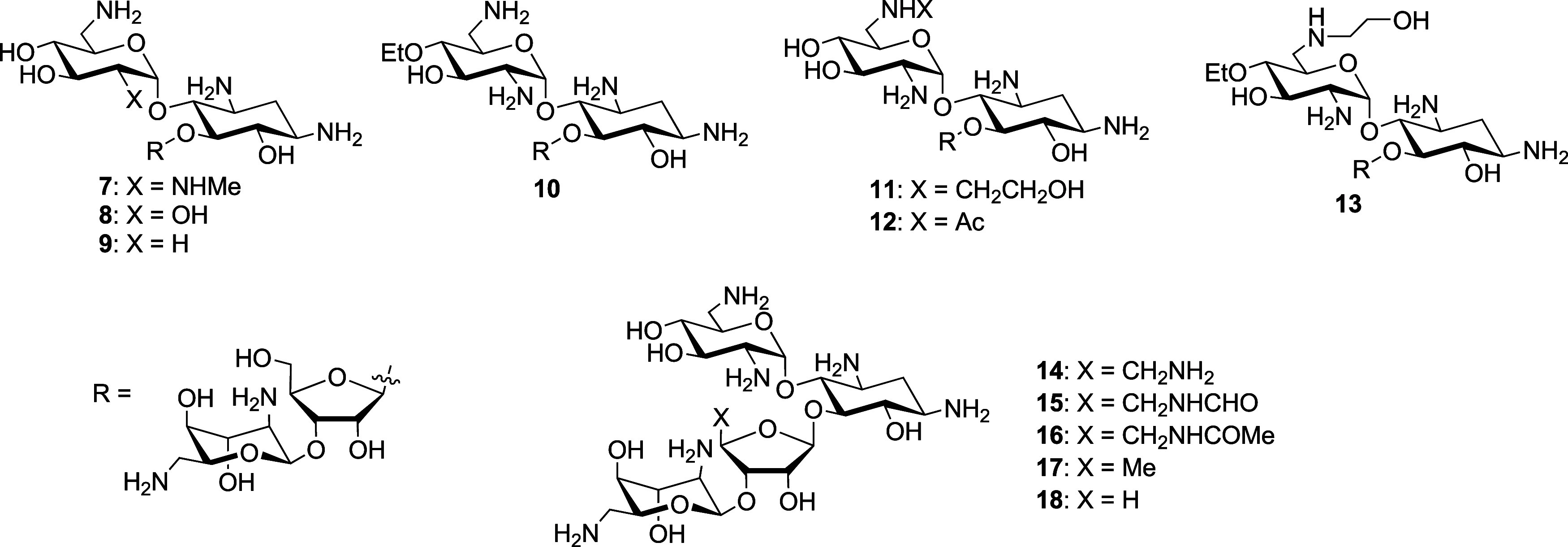
MICs (μg/mL) and Relative Susceptibility of Neomycin Derivatives^a^

			*E. coli* MM294			*E. coli* DH5α			
entry	cmpd	modification(s)	WT *^b^*	ArmA		WT^b^	ArmA	RmtB	susceptibility ratio
1	**7**	2′-NHMe	0.5	1		nd	nd	nd	2
2	**8**	2′–OH	1	2		nd	nd	nd	2
3	**9**	2′-CH_2_	0.5	1		nd	nd	nd	2
4	**10**	4′-OEt	0.5	2		nd	nd	nd	4
5	**11**	6′-NHCH_2_CH_2_OH	0.5	2		nd	nd	nd	4
6	**12**	6′-NHAc	4	32		nd	nd	nd	8
7	**13**	4′-OEt, 6′-NHCH_2_CH_2_OH	4	32		nd	nd	nd	8
8	**14**	5′′-NH_2_	nd	nd		1–2	2	2–4	2
9	**15**	5′′-NHCHO	nd	nd		1	1	1–2	1–2
10	**16**	5′′-NHAc	nd	nd		1	2–4	4–8	2–8
11	**17**	5′′-Me	nd	nd		1	4	4–8	4–8
12	**18**	4′′-CH_2_	nd	nd		1	2–4	4	2–4

a nd = not determined.

b WT refers to strains MM294 and DH5α, respectively, containing the plasmid vector without the RMTase insert.

In the paromomycin series, we studied a series of single modifications at the 2′, 3′ and 4′-positions in ring I, all of which came with susceptibility ratios between 2 and 8 (Table 3, entries 1–4).^37,53^ The 4′-deoxy-4′-propyl paromomycin **23**, otherwise known as propylamycin and the most active member of the series (Table 3, entry 5), revealed a relative susceptibility ratio of 1–2 irrespective of the parent strain, similar to the parental paromomycin. Five modifications, paralleling those made to neomycin, were also made to the 5′′-position of paromomycin, resulting in susceptibility ratios of between 4 and 16 (Table 3, entries 6–10).^42^ Finally, two double modifications were made in which the 4′-deoxy-4′-propyl substitution of propylamycin was complemented with the switching of the 5′′-hydroxy group for either an amino or a formamido group (Table 3, entries 11 and 12).^54^ Both of these doubly modified derivatives came with susceptibility ratios of 2–4, as compared to those of 4–8 for the identical 5′′-substitutions in paromomycin itself.

**Table 3:**
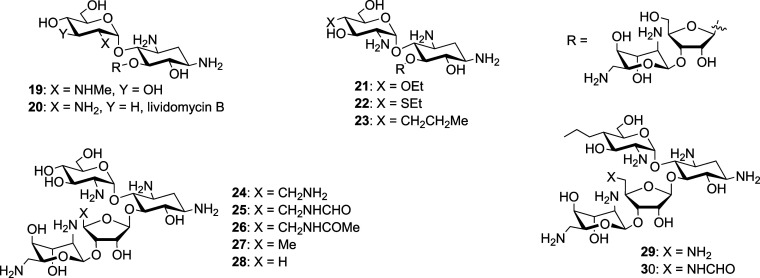
MICs (μg/mL) and Relative Susceptibility of Paromomycin Derivatives^a^

			*E. coli* MM294			*E. coli* DH5α			
entry	cmpd	modification(s)	WT *^b^*	ArmA		WT *^b^*	ArmA	RmtB	susceptibility ratio
1	**19**	2′-NHMe	2–4	16		nd	nd	nd	4–8
2	**20**	3′-deoxy	2–4	8		nd	nd	nd	2–4
3	**21**	4′-OEt	8–16	≥64		nd	nd	nd	>4
4	**22**	4′-SEt	2–4	4–8		nd	nd	nd	2–4
5	**23**	4′-Pr	1	2		0.25–0.5	0.5	0.5	2 *^c^*/1–2 *^d^*
6	**24**	5′′-NH_2_	nd	nd		2	8	8–16	4–8
7	**25**	5′′-NHCHO	nd	nd		1	4	8	4–8
8	**26**	5′′-NHAc	nd	nd		8	nd	128	16
9	**27**	5′′-Me	nd	nd		8	32	32	4
10	**28**	4′′-CH_2_	nd	nd		4	16	16	4
11	**29**	4′-Pr, 5′′-NH_2_	nd	nd		0.5–1	2	2	2–4
12	**30**	4′-Pr, 5′′-NHCHO	nd	nd		0.5–1	2	2	2–4

a nd = not determined.

b WT refers to strains MM294 and DH5α, respectively, containing the plasmid vector without the RMTase insert.

c Susceptibility ratio from the MM294 strains.

d Susceptibility ratio from the DH5α strains.

We have previously noted that when the same structural modification is applied to neomycin and to paromomycin, MIC values are compromised to a greater extent in the paromomycin than in the neomycin series.^42,43^ Comparison of Tables 2 and 3 now reveals that this difference also extends to resistance arising from ribosome modification by the G1405 N7 RMTases. The greater susceptibility of the paromomycin series to AMEs and RMTases than the neomycin series can be attributed to (i) the generally weaker electrostatic interaction of the negatively charged ribosome with the paromomycin series with its five basic amines than with the hexa-amino neomycin series, and (ii) the weaker pseudobase pair interaction of the paromomycin ring I than the neomycin ring I with A1408 in the decoding A site.^55^

In the ribostamycin series only two modifications were investigated involving exchange of the 5′′-hydroxy group for either a formamido or acetamido group (Table 4, entries 1 and 2),^42^ resulting in susceptibility ratios of 2–4 and 4, respectively.

**Table 4:**
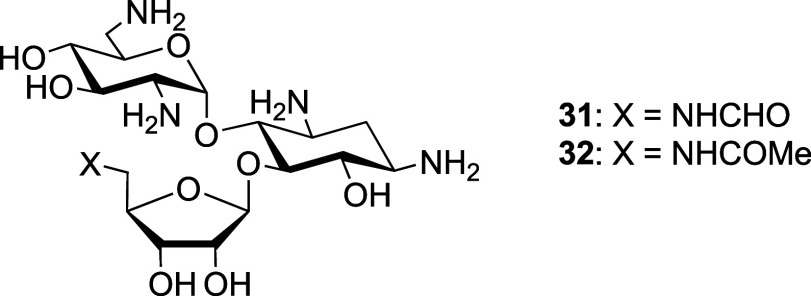
MICs (μg/mL) and Relative Susceptibility of Ribostamycin Derivatives

			*E. coli* DH5α			
entry	cmpd	modification(s)	WT^a^	ArmA	RmtB	susceptibility ratio
1	**31**	5′′-NHCHO	4	8–16	8–16	2–4
2	**32**	5′′-NHAc	16–32	64–128	64–128	4

a WT refers to strain DH5α containing the plasmid vector without the RMTase insert.

Finally, we turned to the apralog and advanced apralog series of compounds^39,40,50^ in which the 5-hydroxy group of the parent apramycin has been appended with a β-d-ribofuranosyl ring, so as to mimic ring III of neomycin, paromomycin, and ribostamycin, with further modifications introduced at the side chain hydroxy group of the ribosyl ring.^56^ All of the apralogs **33**–**38**, except **36**, had activity against the wild type comparable to apramycin (Table 5, entries 1–3, 5, and 6), while **36**, with the additional basic amino group in the 5′′-aminopropylamino substituent, was approximately two times more active against the wild type (Table 5, entry 4). Compounds **39** and **40** are advanced apralogs^40^ carrying an additional aminoethyl chain at the 3-position of the ribosyl ring and either a hydroxy or an amino group at the ribose 5-position, leading to strong activity against the wild type (Table 5, entries 7 and 8). Pertinently, all apralogs and advanced apralogs (Table 5, entries 1–8) had susceptibility ratios of 1–2, i.e., within the technical variance of MIC. These susceptibility ratios indicate that the apralog and advanced apralog series of compounds, if susceptible at all to methylation at G1405 N7, are less so than any of the neomycin, paromomycin, and ribostamycin analogs investigated.

**Table 5:**
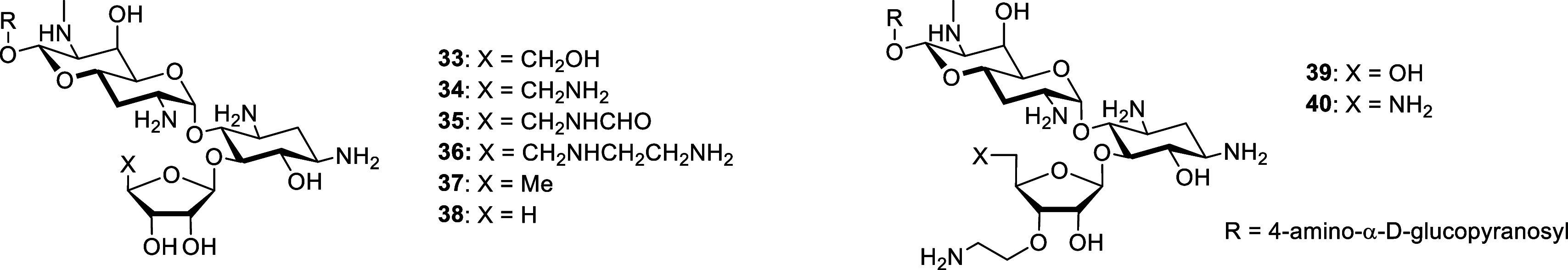
MICs (μg/mL) and Relative Susceptibility of Apramycin Derivatives

			*E. coli* DH5α			
entry	cmpd	modification(s)	WT^a^	ArmA	RmtB	susceptibility ratio
1	**33**	5′′–OH	1–2	1	1–2	1
2	**34**	5′′-NH_2_	2	2	4	1–2
3	**35**	5′′-NHCHO	2–4	2–4	4	1
4	**36**	5′′-NHCH_2_CH_2_NH_2_	0.5–1	0.5	0.5–1	1
5	**37**	5′′-Me	2–4	2	2–4	1
6	**38**	4′′-CH_2_	2	1–2	1–2	1
7	**39**	3′′-OCH_2_CH_2_NH_2_, 5′′–OH	0.5–1	0.5	0.5	0.5–1
8	**40**	3′′-OCH_2_CH_2_NH_2_, 5′′-NH_2_	0.25–1	0.5	0.5	0.5–2

a WT refers to strain DH5α containing the plasmid vector without the RMTase insert.

Overall, modifications introduced to 4,5-AGAs to block AME action, the most common cause of AGA resistance,^1,7–13,24^ may also have the effect of rendering the *E. coli* AGA-ribosome complex in part susceptible to G1405 N7 methylation, i.e., to the action of the RMTases. This effect is modest compared to the very high levels of inhibition of 4,6-AGAs caused by G1405 N7 methylation, but nevertheless, in unfavorable cases, can result in an 8–16-fold loss of activity in strains carrying RMTases compared to the influence of the same modification on the wild type strains. The exceptions to this rule are the apralog and advanced apralog series of compounds obtained by introduction of ribofuranosyl rings to O5 of the 4-monosubstituted AGA apramycin, whose MICs are insensitive to the action of G1405 RMTases. As the sequence of 16S rRNA in the decoding A site is strictly conserved in eubacteria with little, if any, sequence polymorphism^57^ and the effect of G1405 methylation on aminoglycoside resistance has been reported to be identical for all bacterial species where this methylation has been found,^23,25^ we anticipate that the same trends will be seen in other important Gram-negative human pathogens carrying RMTases such as *Klebsiella penumoniae*, *Pseudomonas aeruginosa* , and **Acinetobacter baumannii*.*

To understand the manner by which 4,5-AGA modification gives rise to G1405 RMTase susceptibility we turned to recent high-resolution cryo-EM structures of paromomycin and apramycin with the *E. coli* 70S ribosome that locate critical water molecules for the first time in complete ribosomal subunits.^58,59^ Both the paromomycin (Figure 1A,B, PDB 7K00) and apramycin (Figure 1C,D, PDB 8CGR) structures locate the AGAs in their canonical binding sites but reveal a water-mediated hydrogen bond network connecting the DOS ring and G1405 (Figure 1), related to that apparent in an early structure of paromomycin with an oligoribonucleotide model of the eubacterial ribosomal decoding A site (PDB 1J7T).^60^ More precisely, in the paromomycin-ribosome complex (Figure 1A,B two separate water molecules (blue) are each hydrogen bonded to N1 and O6 in the DOS ring and to G1405 O6. G1405 N7 carries a hydrogen-bonded water molecule (magenta) that, while not itself hydrogen bonded to N1 or O6 in the drug, is located only 3.4 Å from a further water molecule (magenta) that is H-bonded to O6 in the DOS ring (Figure 1). Finally, a further water molecule bridges the gap between O2′′ in the paromomycin ribosyl ring (ring III) and G1405 N7 (Figure 1A). Overall, there are three water-mediated hydrogen bonds between paromomycin and either O6 or N7 of the G1405 guanine ring and two further water molecules, one H-bonded to O6 in the DOS ring and one to G1405 N7, that are in close proximity to each other in the same area between the two molecules.

**Figure 1:**
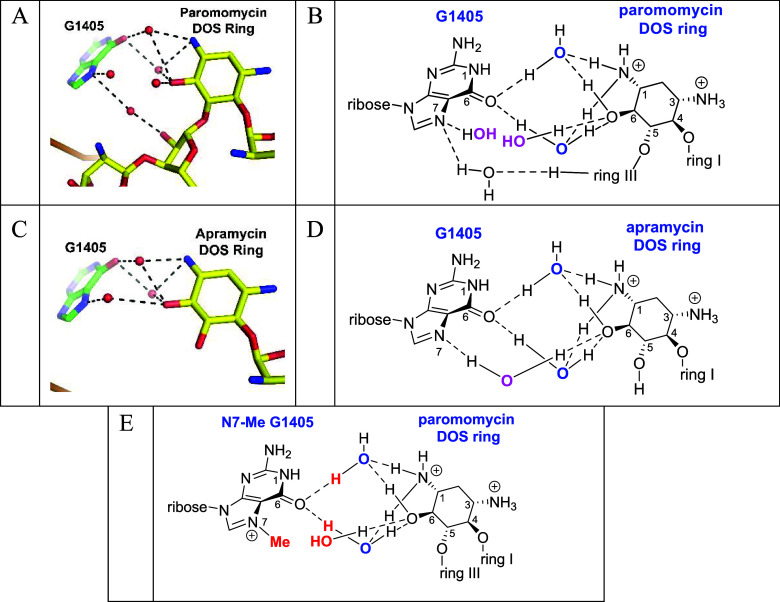
Water-mediated hydrogen bond network linking G1405 to DOS in the *E. coli* 70S ribosome decoding A complex with paromomycin (A, B) and apramycin (C, D) and their hydrophobic and steric destabilization following N7 methylation as illustrated for the paromomycin complex (E).

In the apramycin-ribosome complex (Figure 1C,D), the DOS ring is comparably positioned relative to G1405 as in the paromomycin complex, with N1 and O6 similarly partaking in two water-mediated hydrogen bonds (blue waters) with the guanine O6. Additionally, there is a water (magenta)-mediated hydrogen bond from guanine N7 to the DOS O6. The difference in the water-mediated interaction of G1405 N7 and O6 in the drug DOS ring between the paromomycin and apramycin complexes, two individual but proximal (magenta) water molecules in the former case and one directly bridging (magenta) water in the second instance, presumably reflects the subtly different geometries of the two complexes, which arise from different levels of substitution at the DOS O5 position and different geometries of the pseudobase interactions of ring I with A1408 (not shown).^61,62^

We argue that the water-mediated H-bonding interaction between N1 and O6 of the drug DOS ring and G1405 O6 is an integral component of the overall interaction of both the 4,5-AGAs and apramycin with the drug binding pocket in the bacterial ribosomal decoding A site. A direct consequence of G1405 N7 methylation, illustrated for the paromomycin complex in Figure 1E, is the displacement of the water molecule forming a hydrogen-bonded bridge to the DOS O6 in the apramycin complex and at least one of the two water molecules spanning G1405 N7 and the DOS ring O6 in the paromomycin complex. The second consequence of G1405 N7 methylation and one that is common to both the apramycin and paromomycin complexes is the hydrophobic clash of the new methyl group with the two water molecules bridging guanine, O6, and the DOS ring (Figure 1E) that further destabilizes the drug-ribosome interaction. For the parent 4,5-AGAs and apramycin, this G1405 N7 methylation-induced destabilization is not sufficient to cause a significant reduction in antibacterial activity. However, when modifications are introduced to the AGA framework to overcome AME action a common side effect is some degree of destabilization of the drug-ribosome complex. While it is possible that neither the destabilization caused by G1405 N7 methylation nor that due to AGA modification is sufficient by itself to cause a reduction in activity, the combination of the two is frequently sufficient to do so. The corollary to this argument is that AGA modification by AMEs, designed by nature to destabilize the complex with the ribosome, will also render the compounds more susceptible to RMTase-mediated resistance. The flip side of the argument is that AGA modifications that do not weaken the drug-ribosome interaction, such as the 4′-deoxy-4′-propyl modification in propylamycin **23**, will afford compounds that are only minimally affected by G1405 N7 methylation (Table 3, entry 5). Taking the argument further, AGA modifications that reinforce the drug-ribosome interaction by providing opportunities for greater hydrogen bonding or salt bridges with the drug binding pocket can be expected to yield compounds that are not susceptible to G1405 N7 methylation, with the apralogs and the advanced apralogs providing a prime example of this phenomenon. Alternatively, the differing susceptibilities of the 4,5-AGA and apramycin series complexes to G1405 N7 methylation may arise from the different geometries of the pseudobase interaction of ring I with A1408,^61,62^ which may permit greater adaptability in the apramycin case.

## Conclusions

Methylation of G1405 N7 in the ribosomal decoding A site necessarily gives rise to a hydrophobic clash with two water molecules that bridge N1 and O6 in the DOS ring of the 4,5-AGAs and apramycin and O6 in the guanine residue of G1405. As reflected in MIC values, this hydrophobic interaction is not sufficient to reduce the affinity of the parent drugs for the ribosome to the extent that it results in a reduction in antibacterial activity. However, when the parent drugs are chemically modified to overcome the effect of AMEs, any resulting minor destabilization of the drug-ribosome interaction will combine with that due to G1405 N7 methylation, leading to a composite reduction in affinity that is reflected in the diminution of MIC values. If the AGA modification either has no effect on the drug-ribosome interaction or even augments it, as found for propylamycin and the apralogs, respectively, sensitivity to the G1405 RMTases will not be incurred. Overall, our findings give functional significance to structurally described water-mediated hydrogen bonding from G1405 to N1 and O6 in the 4,5-AGA DOS ring. This contact becomes functionally relevant in the presence of modifications to the AGA framework and G1405 RMTases. Future efforts at next-generation AGAs based on the 4,5-series and/or apramycin should take these factors into account.

### Experimental Section

The chemical synthesis and characterization of all modified AGAs (Table 6) has been described previously.^37,39–43,53,54^

**Table 6:** List of all Modified AGAs Employed and Citations Covering their Synthesis and Characterization

cmpd	ref		cmpd	ref		cmpd	ref
**7**	^43^		**18**	^42^		**30**	^54^
**8**	^43^		**19**	^43^		**31**	^42^
**9**	^43^		**21**	^53^		**32**	^42^
**10**	^41^		**22**	^37^		**33**	^39^
**11**	^41^		**23**	^37^		**34**	^39^
**12**	^41^		**24**	^42^		**35**	^42^
**13**	^41^		**25**	^42^		**36**	^39^
**14**	^42^		**26**	^42^		**37**	^42^
**15**	^42^		**27**	^42^		**38**	^42^
**16**	^42^		**28**	^42^		**39**	^39^
**17**	^42^		**29**	^54^		**40**	^40^

### Recombinant Strains Used and Antibacterial Inhibition Assays

To construct *E. coli* strains expressing *armA* (Genbank accession no. WP_000359986) and *rmtB* (acc. no. WP_012372818) under control of a constitutive promoter, synthetic gene sequences were cloned into a pBR322-derived plasmid backbone as described in ref 64. The plasmids were used to transform chemically competent *E. coli* DH5α cells, resulting in strains expressing *armA* or *rmtB* against an isogenic background. In addition, we tested an *E. coli* strain, obtained from Dr Patrice Courvalin at the Institut Pasteur, expressing *armA* (acc. no. AAP50754) from a natural gene under native promoter control that had been cloned into plasmid pGB2 for transformation of *E. coli* MM294 as described previously.^52^ Strains used in this study have been employed previously as a source of methylated ribosomes for cell-free translation assays^51^ and in the phenotypic profiling of synthetic analogs.^37,40,52^ In addition to conferring high-level resistance to 4,6-AGAs, identical susceptibility ratios for both MM294 ArmA and DH5α ArmA strains were obtained for the 4,5-AGAs, indicating the functional equivalence of the recombinant strains used.

The MICs of synthesized compounds were determined by broth microdilution assays according to CLSI reference methodology M07 ^63^ as described previously.^64^ In brief, compounds were serially 2-fold diluted in cation-adjusted Mueller–Hinton broth, dispensed into 96-well microtiter plates, inoculated with 0.5 × 10^5^ colony forming units in 100 μL per well, and incubated at 37 °C for 16–20 h prior to visual evaluation of bacterial growth. For plasmid maintenance, strains were regularly cultured with antibiotic selection. In each experimental MIC run, and to check for proper RMTase expression, the 4,6-AGA kanamycin was included as a positive control, and paromomycin and apramycin as negative controls.
